# Disseminating and implementing evidence-based practice

**DOI:** 10.3402/ejpt.v4i0.21252

**Published:** 2013-06-06

**Authors:** Jonathan I. Bisson

**Affiliations:** Institute of Psychological Medicine and Clinical Neurosciences, Cardiff University, Cardiff, Wales, UK

**Keywords:** trauma, PTSD, evidence-based, dissemination, implementation

## Abstract

The inconsistent implementation of evidence-based practice has become a significant concern in the traumatic stress field. The European Society for Traumatic Stress Studies (ESTSS) has played a major role in highlighting this issue and has contributed to a number of European initiatives to improve dissemination and implementation. Key initiatives include the introduction of the ESTSS General Certificate in Psychotrauma Psychotraumatology and the European Network for Traumatic Stress (TENTS); these are discussed in this paper.

Just before I left the British Army in 1993, I attended my first European Society for Traumatic Stress Studies (ESTSS) conference in Bergen, Norway. I had never quite experienced anything like it before but have on numerous occasions since. I remember Colin Murray Parkes giving a keynote presentation on bereavement, paying more money for a beer than I had imagined possible and meeting people for the first time who have subsequently become great friends and collaborators. Ironically, I even met Jonathan Shepherd in Bergen who I have subsequently worked closely with in Cardiff.

I have always felt at home with the ESTSS; it was and is a welcoming, multidisciplinary society in which everyone shares a passion to help those exposed to traumatic events. It has been a perfect setting for me to combine my interest in both clinical practice and research and, increasingly, to disseminate and implement an evidence-based approach to practice. As a member of ESTSS, I was honored to be asked to be the European representative on the first treatment guidelines committee of the International Society of Traumatic Stress Studies; I led on the guidelines for *Psychological Debriefing* (Bisson, McFarlane, & Rose, 2000). This experience and concerns around non-evidence-based practice increased my appetite for systematically reviewing and meta-analyzing evidence within the traumatic stress field. As a result, I co-chaired the Guideline Development Group for the UK's NICE guideline on the management of PTSD (National Collaborating Centre for Mental Health [NCCMH], 2005) and have co-authored four published Cochrane reviews in the field.

I joined the ESTSS Board in 2001 at the Edinburgh conference and have fond memories of wearing a kilt for the first (and possibly the last) time in my life.

When I became President in 2007, Berthold Gersons had already undertaken a lot of work to transform the ESTSS and I was able to continue with that work. I remember several tense meetings with the presidents of different societies but by positively working together, we managed to find a way forward. It was great to see the creation of new societies in the Netherlands, United Kingdom, Poland, and Georgia and a large expansion of the ESTSS membership.

## ESTSS General Certificate in Psychotraumatology

In addition to driving forward the transition of ESTSS, I was keen to develop the Society's work in the dissemination and implementation of evidence-based practice during my Presidency. We introduced the ESTSS General Certificate in Psychotraumatology; an initiative that I continue to believe has the potential to be developed further.

The ESTSS Board approved the introduction of the ESTSS General Certificate in Psychotraumatology in December 2007. All members of the ESTSS are eligible to register for the certificate program subject to a one-off fee to cover the administration of the certification process. To complete the Certificate, individuals must attend a 1-day ESTSS-approved introductory workshop and then 4 days of other approved workshops. Satisfactory completion of a test for each workshop attended and completion of all workshops within 3 years of attendance at the first qualifying workshop are the final requirements. A wide range of workshops are eligible for the Certificate allowing those undertaking it to develop a strong grounding in psychotraumatology.

There are now an increasing number of individuals from across Europe who have been awarded the Certificate, and it has attracted people to the Society who may not have become involved otherwise. I consider the Certificate an excellent way to disseminate knowledge in an evidence-based manner.

## The European Network for Traumatic Stress and the development of evidence-based guidelines

In parallel with the development of the Certificate, members of the ESTSS Board along with some other interested parties created a team, led by Miranda Olff, to apply for European Union (EU) funding to develop European guidance on psychosocial responses following traumatic events. The European Network for Traumatic Stress (TENTS) was formed (Witteveen et al., 2012; see also Olff, 2013).

The initial project identified an absence of high-quality research to inform the development of evidence-based guidelines and resulted in a Delphi process being undertaken to develop a consensus of items that should be included in a psychosocial care guideline following disasters by an independent group of 106 experts from 25 different countries (Bisson et al., 2010). The process resulted in the production of the TENTS guidelines for psychosocial care following disasters and major incidents (TENTS, 2010). The guidelines state that all aspects of psychosocial care should only be provided with full consideration of the individuals’ wider social environment, especially their families and communities. The 6 sections and 54 recommendations cover planning, preparation, and management; general components of the response; and specific components to be included at particular phases of the response.

## TENTS Training and Practice

The success of the TENTS project resulted in further EU funding being awarded to consolidate the initial work through TENTS-Training and Practice (TENTS-TP). The TENTS guidelines together with guidance produced by NATO (2008) facilitated the development of the evidence-based TENTS-TP curriculum to train health and social care professionals to help individuals affected by traumatic events (TENTS, 2012). Another Delphi process was followed to develop a *Train the Trainer* program (Pearce et al., 2012), which is now being disseminated through Europe. To date, there are 35 participating countries, there have been 31 workshops and 462 potential new trainers have been trained. It is great that TENTS has been formally integrated into ESTSS, which will hopefully allow it to further develop.

I continue to feel very attached to ESTSS and remain committed to helping it to fully exploit its key role in disseminating and implementing evidence-based practice in the traumatic stress field across Europe and beyond.
